# A β-glucosidase hyper-production *Trichoderma reesei* mutant reveals a potential role of cel3D in cellulase production

**DOI:** 10.1186/s12934-016-0550-3

**Published:** 2016-09-01

**Authors:** Chengcheng Li, Fengming Lin, Yizhen Li, Wei Wei, Hongyin Wang, Lei Qin, Zhihua Zhou, Bingzhi Li, Fugen Wu, Zhan Chen

**Affiliations:** 1 State Key Laboratory of Bioelectronics, School of Biological Science and Medical Engineering, Southeast University, Nanjing, 210096 China; 2 State Key Laboratory of Medical Genomics, Ruijin Hospital, Shanghai Jiao Tong University School of Medicine, Shanghai, China; 3 Key Laboratory of Synthetic Biology, Institute of Plant Physiology and Ecology, Shanghai Institutes for Biological Sciences, Chinese Academy of Sciences, Shanghai, 200032 China; 4 Key Laboratory of Systems Bioengineering (Ministry of Education), School of Chemical Engineering and Technology, Tianjin University, Weijin Road 92, Nankai District, 300072 People’s Republic of China; 5 Department of Chemistry, University of Michigan, 930 North University Avenue, Ann Arbor, MI 48109 USA; 6 37 Jinxianghe Road, Xuanwu District, Nanjing, 210008 Jiangsu Province China

**Keywords:** *Trichoderma reesei*, Cellulase, β-Glucosidase, Hyper-production, Carbon catabolite repression, Cel3d

## Abstract

**Background:**

The conversion of cellulose by cellulase to fermentable sugars for biomass-based products such as cellulosic biofuels, biobased fine chemicals and medicines is an environment-friendly and sustainable process, making wastes profitable and bringing economic benefits. *Trichoderma reesei* is the well-known major workhorse for cellulase production in industry, but the low β-glucosidase activity in *T. reesei* cellulase leads to inefficiency in biomass degradation and limits its industrial application. Thus, there are ongoing interests in research to develop methods to overcome this insufficiency. Moreover, although β-glucosidases have been demonstrated to influence cellulase production and participate in the regulation of cellulase production, the underlying mechanism remains unclear.

**Results:**

The *T. reesei* recombinant strain TRB1 was constructed from *T. reesei* RUT-C30 by the T-DNA-based mutagenesis. Compared to RUT-C30, TRB1 displays a significant enhancement of extracellular β-glucosidase (BGL1) activity with 17-fold increase, a moderate increase of both the endoglucanase (EG) activity and the exoglucanase (CBH) activity, a minor improvement of the total filter paper activity, and a faster cellulase induction. This superiority of TRB1 over RUT-C30 is independent on carbon sources and improves the saccharification ability of TRB1 cellulase on pretreated corn stover. Furthermore, TRB1 shows better resistance to carbon catabolite repression than RUT-C30. Secretome characterization of TRB1 shows that the amount of CBH, EG and BGL in the supernatant of *T. reesei* TRB1 was indeed increased along with the enhanced activities of these three enzymes. Surprisingly, qRT-PCR and gene cloning showed that in TRB1 β-glucosidase cel3D was mutated through the random insertion by AMT and was not expressed.

**Conclusions:**

The *T. reesei* recombinant strain TRB1 constructed in this study is more desirable for industrial application than the parental strain RUT-C30, showing extracellular β-glucosidase hyper production, high cellulase production within a shorter time and a better resistance to carbon catabolite repression. Disruption of β-glucosidase cel3D in TRB1 was identified, which might contribute to the superiority of TRB1 over RUT-C30 and might play a role in the cellulase production. These results laid a foundation for future investigations to further improve cellulase enzymatic efficiency and reduce cost for *T. reesei* cellulase production.

**Electronic supplementary material:**

The online version of this article (doi:10.1186/s12934-016-0550-3) contains supplementary material, which is available to authorized users.

## Background

Cellulose is abundantly presented in nature and in waste materials in the form of wood, grass, leaves, agricultural wastes (straw, husk, corn cob, and begass et al.), food processing wastes, timber wastes, municipal wastes and so on. Cellulose is renewable, inexpensive and environment-friendly by deriving its carbon from the air instead of petroleum or natural gas. Therefore, bioconversion of cellulose by cellulase to fermentable sugars which is further utilized by microorganisms to produce cellulosic biofuels, biobased fine chemicals and medicines, is environment-friendly and sustainable by making good use of waste and bringing economic efficiency. Cellulase is a complex, extracellular enzyme mixture, mainly consisting of three synergistic enzymes participating in the degradation of cellulose: endoglucanase (EG, EC3.2.1.4), exoglucanase (or cellobiohydrolase, CBH, EC 3.2.1.91), and β-glucosidase (BGL, EC 3.2.1.21) [1]. EG randomly hydrolyzes the internal glycosidic linkages in the non-crystalline area of cellulose, mainly producing cellodextrin and oligosaccharides. CBH liberates cellobiose units from either the reducing or nonreducing ends of cellulose chain. Then, BGL hydrolyzes cellobiose and oligosaccharides to release fermentable d-glucose [1].

Currently, the cellulase used in industry is produced mainly from bacteria and fungi (*Penicllium*, *Aspergillus*, and *Trichoderma*) [2]. Among them, the filamentous fungus *Trichoderma reesei* is the important industrial workhorse, because it has remarkable ability to produce cellulase in quantities exceeding 100 g/L [3]. However, *T. reesei* has low β-glucosidase activity, which reduces efficiency in biomass degradation and compromises its industrial application [4]. Under cellulase-inducing conditions, the production of secreted β-glucosidase comprises only about 1 % of the total *T. reesei* cellulase [5]. Thus, there are ongoing interests in research to increase β-glucosidase amount in the cellulase complex from *T. reesei*. The supplementation of β-glucosidase produced by other fungi to the *T. reesei* cellulase preparations has been employed to increase the enzyme efficiency of hydrolyzing cellulosic substrates [6, 7]. Meanwhile, the construction of a single recombinant *T. reesei* that produces the full set of saccharifying enzymes in optimum amount, including BGL, represents another approach to reduce processing cost. Genetic engineering has been successfully utilized to increase the BGL activity in *T. reesei*, as well as the cellulase production. The activity of β-glucosidase in *T. reesei* has been enhanced by heterologous expression of exotic β-glucosidases from other fungi, such as *Penicillium decumbens* [8], *Aspergillus aculeatus* [9, 10], and *Periconia* sp. [11]. Using strong artificial cellobiohydrolase 1 promoter to express *T. reesei* extracellular β-glucosidase (BGL1) led to 3.7-fold increase in β-glucosidase activity of *T. reesei* [12]. In spite of all the genetic efforts which have been done, there is still no available *T. reesei* strain which could produce cellulase with optimal amounts for different components.

On the basis of the *T. reesei* genome database v.2.0 (http://www.genome.jgipsf.org/Trire2/Trire2.home.html), at least 10 genes encoded β-glucosidase isozymes have been identified: cel1A, cel1B, cel3A, cel3B, cel3C, cel3D, cel3E, cel3F, cel3G and cel3H. Among them, cel3A (bgl1) is the major extracellular β-glucosidase in cellulase production [5], while cel1A (bgl2) [13] and cel1B [14] are intracellular. In addition, it is presumed that cel3B, cel3E, cel3F, cel3G and cel3H are extracellular, while cel3C, cel3D and cel3H are intracellular [15]. The involvement of β-glucosidases in cellulase production has been studied by characterization of mutants with knockout of different β-glucosidases. Single deletion of cel3A caused a lag phase of the cellulase induction by cellulose in *T. reesei* [16]. Single deletion *T. reesei* mutants of cel1A or cel1B exhibited a delay in the induction of cellulase gene but unaffected cellulase production by cellulose, while these mutants displayed normal induction of cellulase genes but increased cellulase production by cellobiose [14]. Moreover, cel1A and cel1B are required for the cellulase induction by lactose in *T. reesei* [17]. An amino acid substitution V409F in enzyme Cel1A was found in the cellulase hyper-producing mutant PC-3-7 [18], which is responsible for the enhanced cellulase production in PC-3-7 using cellobiose as the carbon source [19]. Disruption of the transcription factor BglR in strain PC-3-7, which regulates β-glucosidase genes except cel3A in *T. reesei*, resulted in elevated cellulase production on cellobiose [20]. Apparently, the effect of cel3A, cel1A and cel1B on cellulase production varied as the corresponding mutants are grown on different carbon sources. Although all these current studies have shown that β-glucosidases take part in cellulase biosynthesis regulation, Knowledge with respect to the underlying mechanism is lack.

In the present study, the *T. reesei* recombinant strain TRB1 was derived from *T. reesei* RUT-C30 by the T-DNA-based mutagenesis. Compared to RUT-C30, TRB1 displays a substantial enhancement of extracellular β-glucosidase activity, a modest increase of both the endoglucanase activity and the exoglucanase activity, a slight increment of the total filter paper activity, and a faster cellulase induction. This superiority of TRB1 over RUT-C30 is independent on carbon sources. Furthermore, the underlying mechanism for the β-glucosidase hyper-production in TRB1 was investigated.

## Results

### Recombinant *T. reesei* strain TRB1 shows β-glucosidase hyper-production and faster cellulase induction

**Fig. 1:**
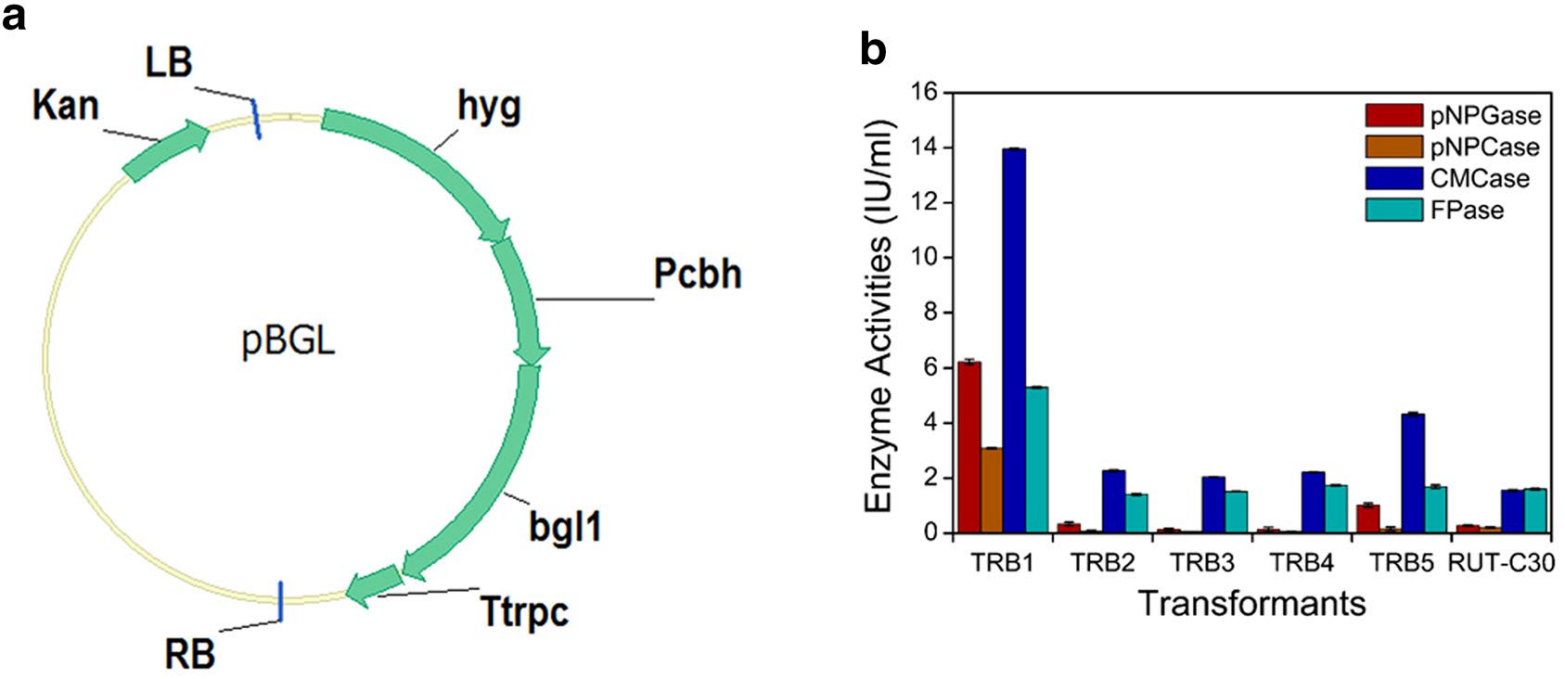
**a** Schematic illustration of the plasmid pBGL. *Kan* kanamycin resistance; *LB* left border of binary vector; *RB* right border of binary vector; *Pcbh* a modified CBH promoter [21]; *Ttrpc Aspergillus nidulans* trpC terminator; *bgl1 T. reesei* bgl1 gene; *hph* hygromycin B phosphotransferase gene. **b** Cellulase activity of *T. reesei* RUT-C30 and the five recombinant *T. reesei* strains: TRB1, TRB2, TRB3, TRB4 and TRB5, using cellulose as the carbon source. *pNPGase* the β-glucosidase activity; *pNPCase* the CBH activity; *CMCase* the CMC activity; *FPase* the filter paper activity. The *error bars* indicate the standard deviation of three replicates, though in some cases they are too small to see on the graphical scale being used

Gene bgl1 (containing an C-terminal 6-histidine tag) from *T. reesei* was cloned into plasmid pDht/sk under a modified CBH1 promoter [21], resulting in the plasmid construction pBGL (Fig. 1a). pBGL was transformed into *T. reesei* RUT-C30 by AMT method and five *T. reesei* transformants were obtained: TRB1, TRB2, TRB3, TRB4 and TRB5. Surprisingly, we found that the cellulase production of TRB1 was much higher than other recombinant strains and strain RUT-C30 on day 3 cultivation using cellulose as the carbon source (Fig. 1b). Compared to the parental strain RUT-C30, the pNPGase activity (the β-glucosidase activity), the CMCase activity (the CMC activity), the pNPCase activity (the CBH activity) and the FPase activity (the filter paper activity) in strain TRB1 on day 3 were increased by 23-, 8.7-, 3.3- and 22.4-fold, respectively.

**Fig. 2:**
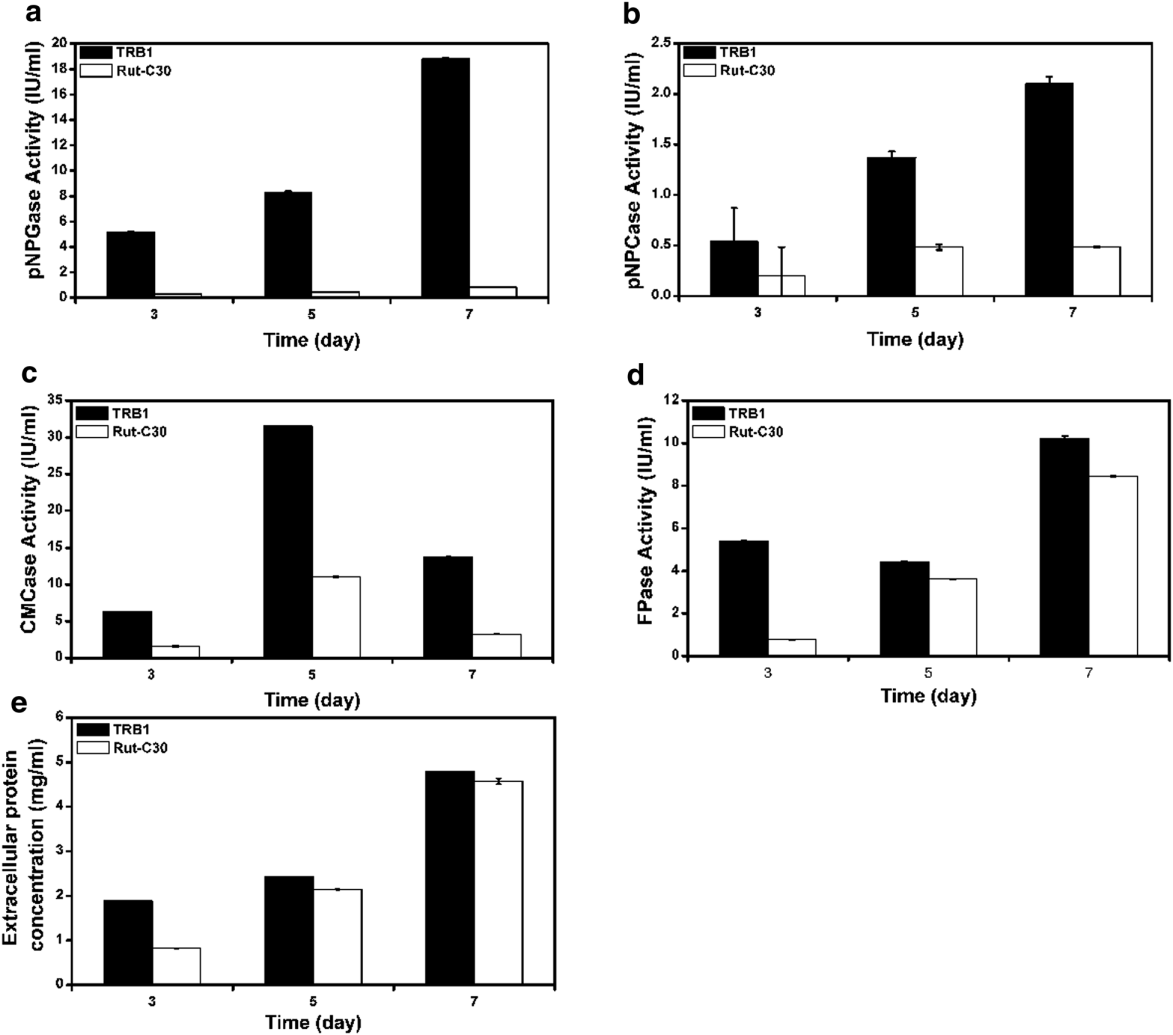
Cellulolytic enzyme activities in the culture supernatants of *T. reesei* Rut-C30 and TRB1 which were collected on day 3, 5, 7 on 2 % microcrystalline cellulose for 7 days. The activities of pNPGase (the BGL activity), pNPCase (the CBH activity), CMCase (the CMC activity), and FPA (the filter paper activity), and the protein concentration are listed in **a**–**e** respectively. The *error bars* indicate the standard deviation of three replicates, though in some cases they are too small to see on the graphical scale being used

In details, the time course of various enzymes’ activities and total protein concentration in the supernatant of strain TRB1 was determined (Fig. 2). The recombinant strain TRB1 showed an excellent β-glucosidase activity throughout the whole cellulase production process, which reached the highest activity of 19 IU/mL on day 7 with a 27-fold increase (Fig. 2a). Similar to β-glucosidase activity, the CBH activity in TRB1 kept rising from day 3 to 7 with the maximum activity of about 2.0 IU/mL, and was 4.4-fold higher than that in strain RUT-C30 (Fig. 2b). The CMCase activity of both strain RUT-C30 and strain TRB1 peaked on day 5, with the maximum activity of 10 and 30 IU/mL respectively (Fig. 2c). The CMC activity was increased by 4.2-fold in TRB1 on day 7 (Fig. 2c). Therefore, TRB1 greatly outperforms strain RUT-C30 during the whole time course in terms of the pNPGase activity, the CMCase activity and the pNPCase activity. However, the FPase activity which represents the total filter paper enzyme activity was only slightly increased with 11 % (Fig. 2d). This may be due to the fact that the total protein concentration of the supernatant of strain TRB1 is almost the same as that of strain RUT-C30 (Fig. 2e). More discussions on this issue will be presented below. In addition, high cellulase activity in the supernatant of strain TRB1 was detected even at the early cultivation stage (day 3), indicating a faster cellulase induction and production in strain TRB1 in comparison with the parental strain RUT-C30 (Fig. 2d).

### The outperformance of strain TRB1 is independent of carbon sources

**Fig. 3:**
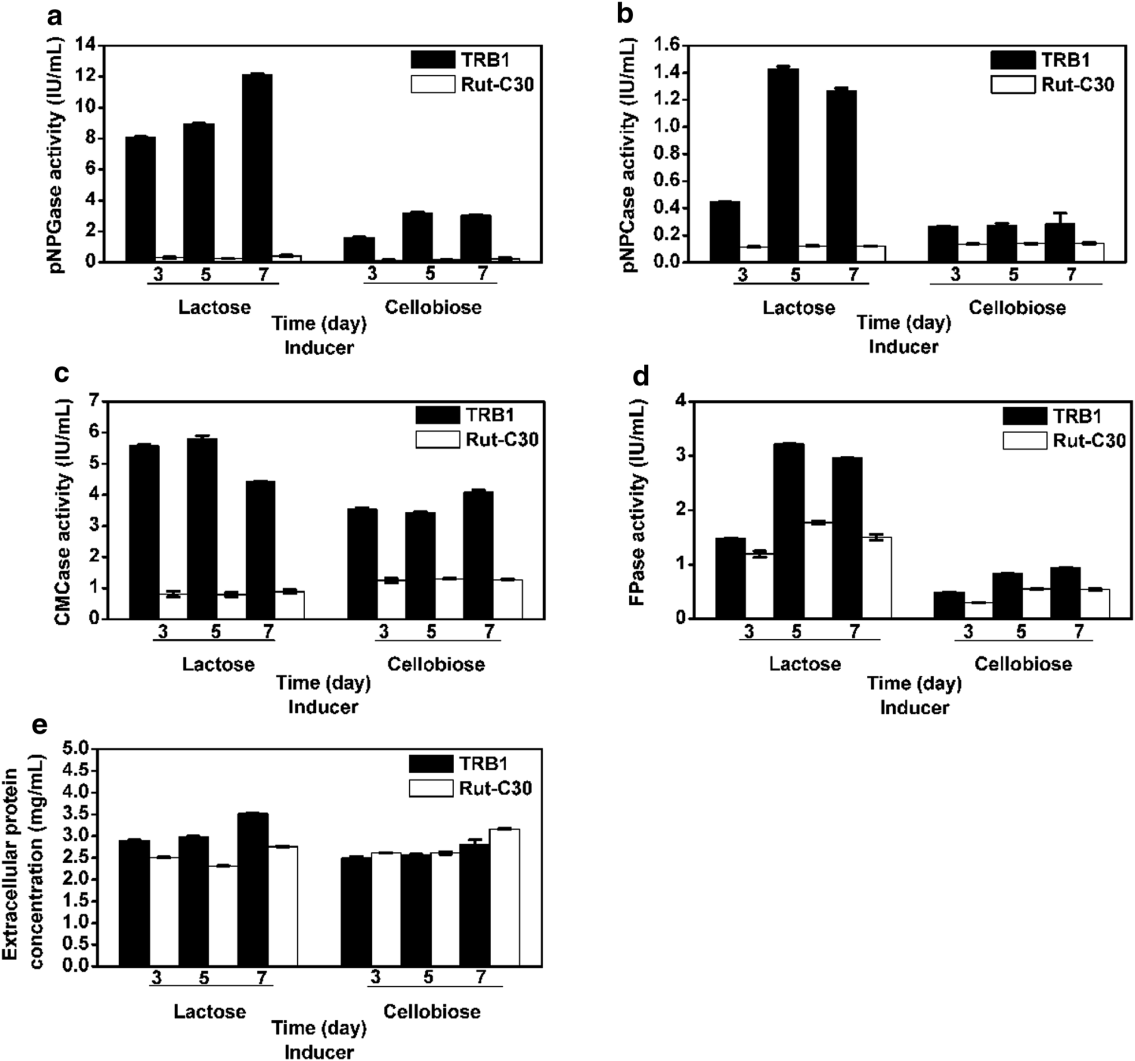
Cellulolytic enzyme activities in the extracellular culture supernatant of *T. reesei* RUT-C30 and TRB1 which were collected on day 3, 5, 7 on cellobiose and lactose for 7 days. The activities of pNPGase (the BGL activity), pNPCase (the CBH activity), CMCase (the CMC activity), and FPA (the filter paper activity), and the protein concentration are listed in **a**–**e** respectively. The *error bars* indicate the standard deviation of three replicates, though in some cases they are too small to see on the graphical scale being used

The performance of *T.reseei* mutants often rely heavily on what kind of carbon source is available for them and sometimes various results were obtained when using different carbon sources [14, 22]. Hence, we wonder whether the outperformance of recombinant *T. reesei* strain TRB1 in this study is carbon source dependent. To this end, the pNPGase, pNPCase, CMCase, FPase activities and protein concentration of strain *T. reesei* RUT-C30 and TRB1 were analyzed in time course when grown on media containing cellobiose, lactose, galactose and sucrose as the sole carbon source. Very poor cellulase induction was observed for both strain RUT-C30 and strain TRB1 on either galactose or sucrose (data not shown). For both *T. reesei* RUT-C30 and TRB1, lactose is not a good cellulase inducer as cellulose, but better than cellobiose (Fig. 3). Most interestingly, the superiority of TRB1 over RUT-C30 remained on both lactose and cellobiose, resembling cellulase induction and production by cellulose: a significant enhancement of the pNPGase activity (Fig. 3a), a moderate increase of the CMCase activity (Fig. 3b) and the pNPCase activity (Fig. 3c), a minor increasement of FPase activities (Fig. 3d), unaffected extracellular protein production ability (Fig. 3e) and high cellulase activity at the early cultivation stage (Fig. 3). The maximal β-glucosidase activity of TRB1 induced by lactose was 12 IU/mL, as high as that by cellulose, while it was only 3.0 IU/mL by cellobiose (Fig. 3a). These results suggested that the observed outperformance of TRB1 over RUT-C30 was independent of carbon sources, a flexibility enabling its wide use in industry.

### *Trichoderma reesei* TRB1 shows a better resistance to carbon catabolite repression than *T. reesei* RUT-C30

**Fig. 4:**
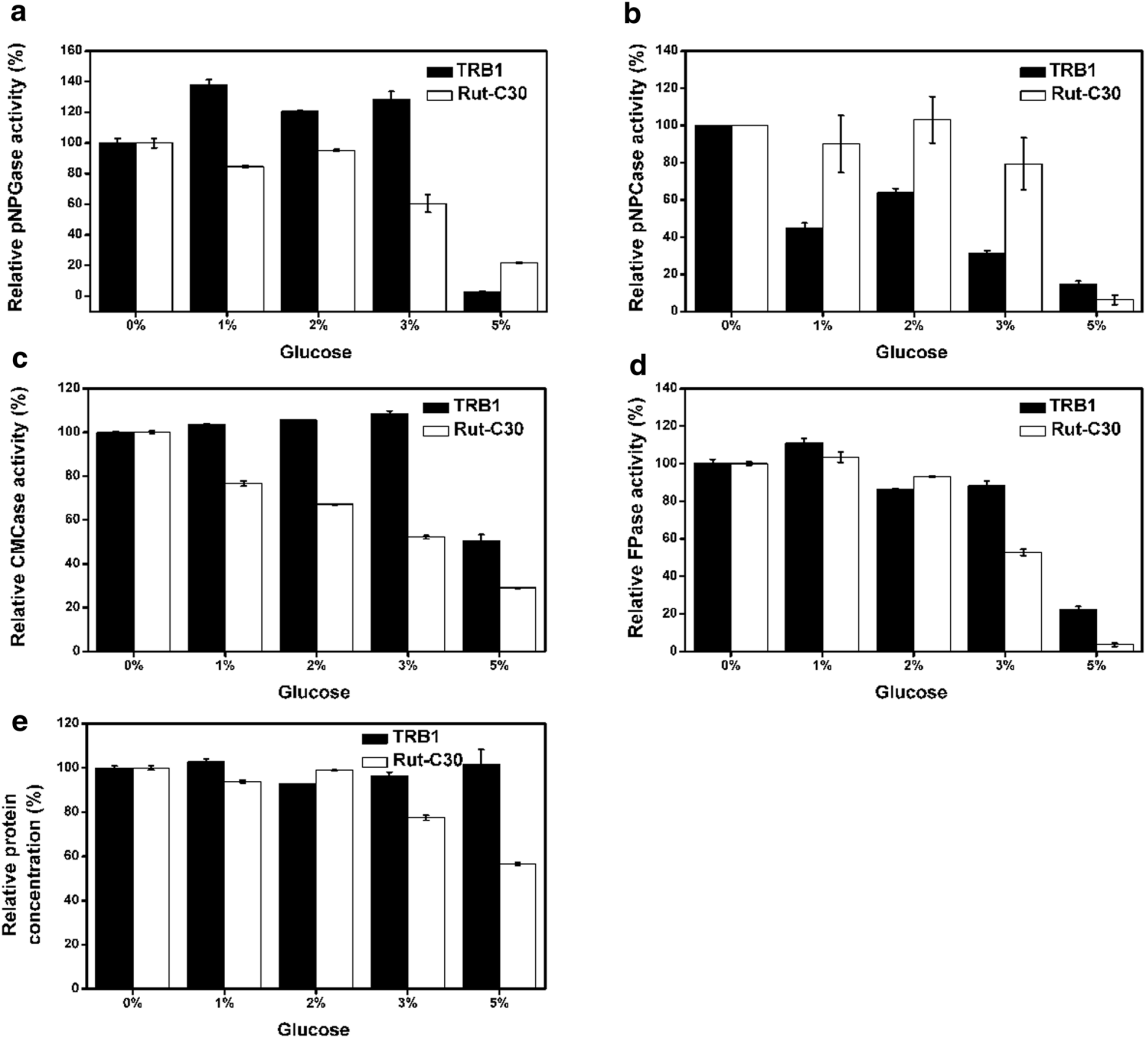
Effects of glucose on the cellulolytic enzyme activities of *T. reesei* RUT-C30 and TRB1 which were induced on cellulose. The activities of pNPGase (BGL activity), pNPCase (CBH activity), FPA (Filter paper activity), CMCase (CMC activity) on day 7, and the protein concentration are listed in **a**–**e** respectively. The *error bars* indicate the standard deviation of three biological replicates. The activities of pNPGase (BGL activity), pNPCase (CBH activity), FPA (Filter paper activity), CMCase (CMC activity) on day 7 in the absence of glucose are arbitrarily assigned as 100 % in TRB1 and RUT-C30 individually and the corresponding activities are referred to Fig. 1. The *error bars* indicate the standard deviation of three replicates, though in some cases they are too small to see on the graphical scale being used

The resistance of strain TRB1 to carbon catabolite repression (CCR) by glucose was evaluated in flask cultures with 2 % avicel plus different concentrations of glucose (0, 1, 2, 3, 5 and 10 %). The time course of the activities of pNPGase, pNPCase, CMCase, pNPXase and FPase is shown in Fig. 4. The CMCase activity in RUT-C30 is decreased gradually as the concentration of glucose in present is increased, while the CMCase activity in TRB1 was not affected by the present glucose with no more than 3 % and was rescued sharply by ~50 % in the presence of 5 % glucose (Fig. 4c). The pNPCase activity of TRB1 is depressed noticeably by 50 % in the presence of glucose. But the corresponding pNPCase activity in concrete numerical values 2.7, 1.2, 1.7 and 0.4 IU/ mL in the presence of 0, 1, 2 and 5 % glucose respectively, is still higher than that of RUT-C30 (1.0, 0.9, 1.0 and 0.06 IU/mL) which is unaffected by glucose when the glucose concentration is no more than 3 % (Fig. 4b). When the glucose concentration is beyond 3 %, all components of cellulase production in TRB1 and RUT-C30 reduced sharply (Fig. 4), indicating a strong CCR effect exits at 5 % or higher glucose concentration. Since RUT-C30 is evolved from *T. reesei* QM6a through three rounds of mutagenesis followed by a screening for alleviated carbon catabolite repression and high cellulase activity [23], so it is not surprising to see that RUT-C30 exhibited a good resistance to glucose. However, TRB1 exhibited even better resistance to glucose. Especially, the pNPGase activity of TRB1 even increased by 20–40 % in the presence of 1–3 % glucose in comparison with the absence of glucose, which is not observed in RUT-C30 (Fig. 4a). The presence of glucose did not affect the extracellular protein concentration of TRB1 no matter what the glucose concentration is, but reduced that of RUT-C30 with the concentration of more than 2 % (Fig. 4e). No cellulase production was detected in TRB1 when using glucose as the sole carbon source (data not shown). Here we demonstrated that TRB1 displays a better resistance to carbon catabolite repression than RUT-C30.

### Biomass saccharification

**Fig. 5:**
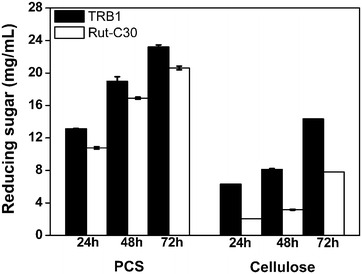
Saccharification of PCS and microcrystalline cellulose by *T. reesei* RUT-C30 and TRB1 using the equal culture supernatants. The *error bars* indicate the standard deviation of three biological replicates. The *error bars* indicate the standard deviation of three replicates, though in some cases they are too small to see on the graphical scale being use

The hydrolysis efficiency of crude cellulase preparation produced by strain RUT-C30 (CC-RUT) and TRB1 (CC-TRB1) was tested using PCS and microcrystalline cellulose as substrates (Fig. 5). Both CC-RUT and CC-TRB1 showed higher hydrolysis ability on PCS than on microcrystalline cellulose. CC-TRB1 released more reducing sugar than CC-RUT on both PCS and cellulose during the whole hydrolysis incubation time. At 72 h, CC-TRB1 released 23.2 and 14.3 mg/mL reducing sugar on PCS and cellulose respectively, while CC-RUT yielded 20.6 and 7.8 mg/mL reducing sugar individually. There is about 11 and 45 % increase in the hydrolysis efficiency of CC-TRB1 on PCS and cellulose respectively as compared to CC-RUT. Clearly, the high concentration of β-glucosidase in CC-TRB1 helps improve its biomass saccharification ability.

### Characterization of secretome of *T. reesei* TRB1 by SDS-PAGE and MUG-zymogram analysis

**Fig. 6:**
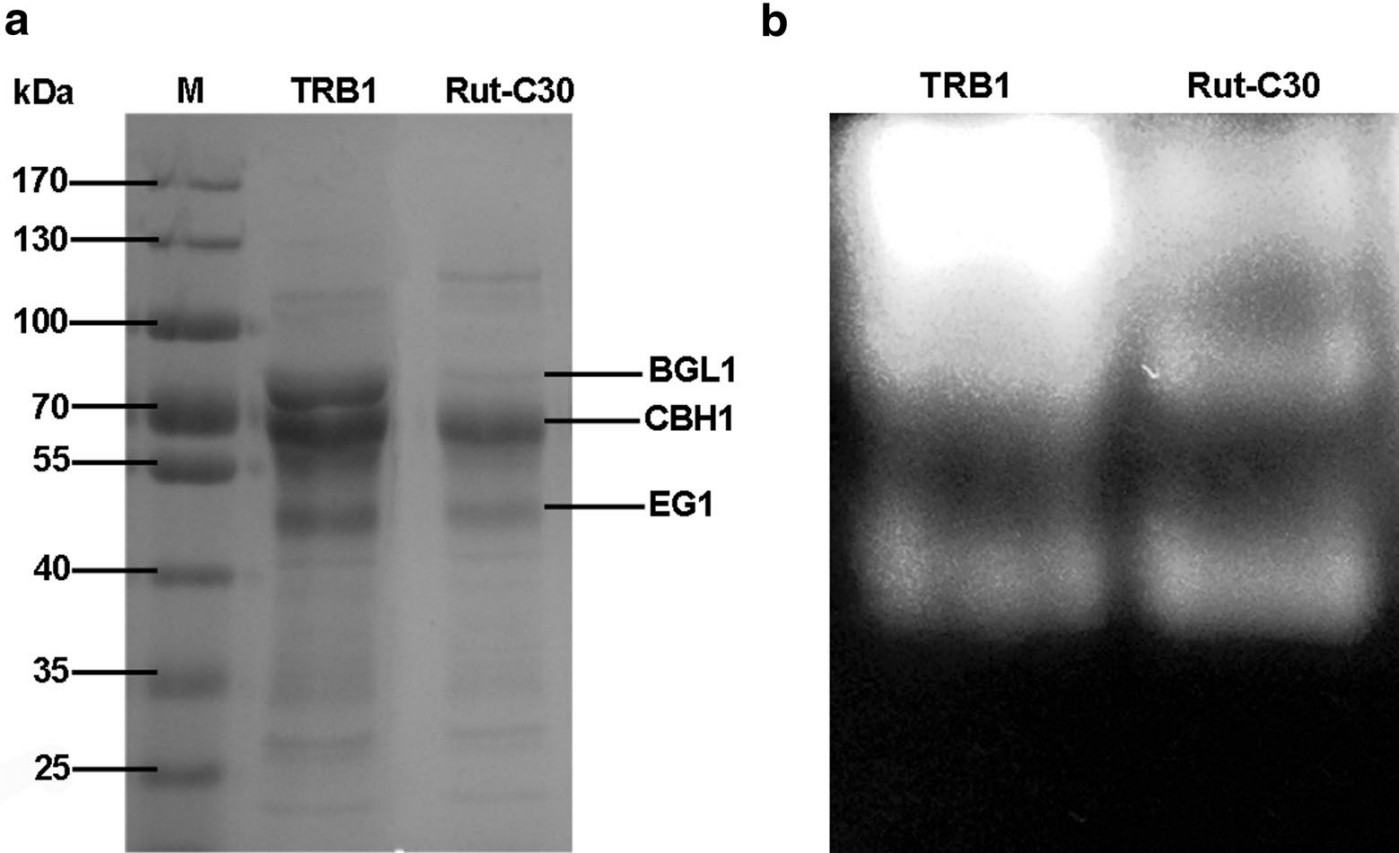
**a** SDS-PAGE analysis of secretome of *T. reesei* RUT-C30 and TRB1 grown on cellulose for 1 week. *Lane M* DNA molecular weight marker. Equal protein loading was used. Based on the molecular weight, the estimated position for BGL, CBH and EG1 was labeled, respectively. **b** Identification of β-glucosidases in the culture supernatant of *T. reesei* RUT-C30 and TRB1 by MUG-zymogram assay

The supernatants from *T. reesei* RUT-C30 and TRB1 cultured in TMM with 2 % avicel on day 7 were profiled by SDS-PAGE. When equal protein loading was used, the protein profile secreted by TRB1 is different from that by the parent strain RUT-C30 (Fig. 6a). TRB1 secreted more BGL1, CBH1 and EG1 than RUT-C30, which is consistent with the above observed increases of the pNPGase, CMCase and pNPCase activities and can be used to explain such increases. Furthermore, identification of β-glucosidases in the supernatant of both *T. reesei* RUT-C30 and TRB1 grown on cellulose was done by the MUG-zymogram assay (Fig. 6b). We observed three bands from each sample on the MUG gel, probably representing three types of β-glucosidases. Notably, the first upper band was enhanced substantially in the supernatant of *T. reesei* TRB1, compared to that of RUT-C30. It seems that the elevated extracellular β-glucosidase activity we observed in TRB1 is due to the increased amount of secreted β-glucosidase, not due to more types of β-glucosidases that were secreted into the supernatant of TRB1.

### The expression of cel3D is disrupted in recombinant *T. reesei* TRB1

**Fig. 7:**
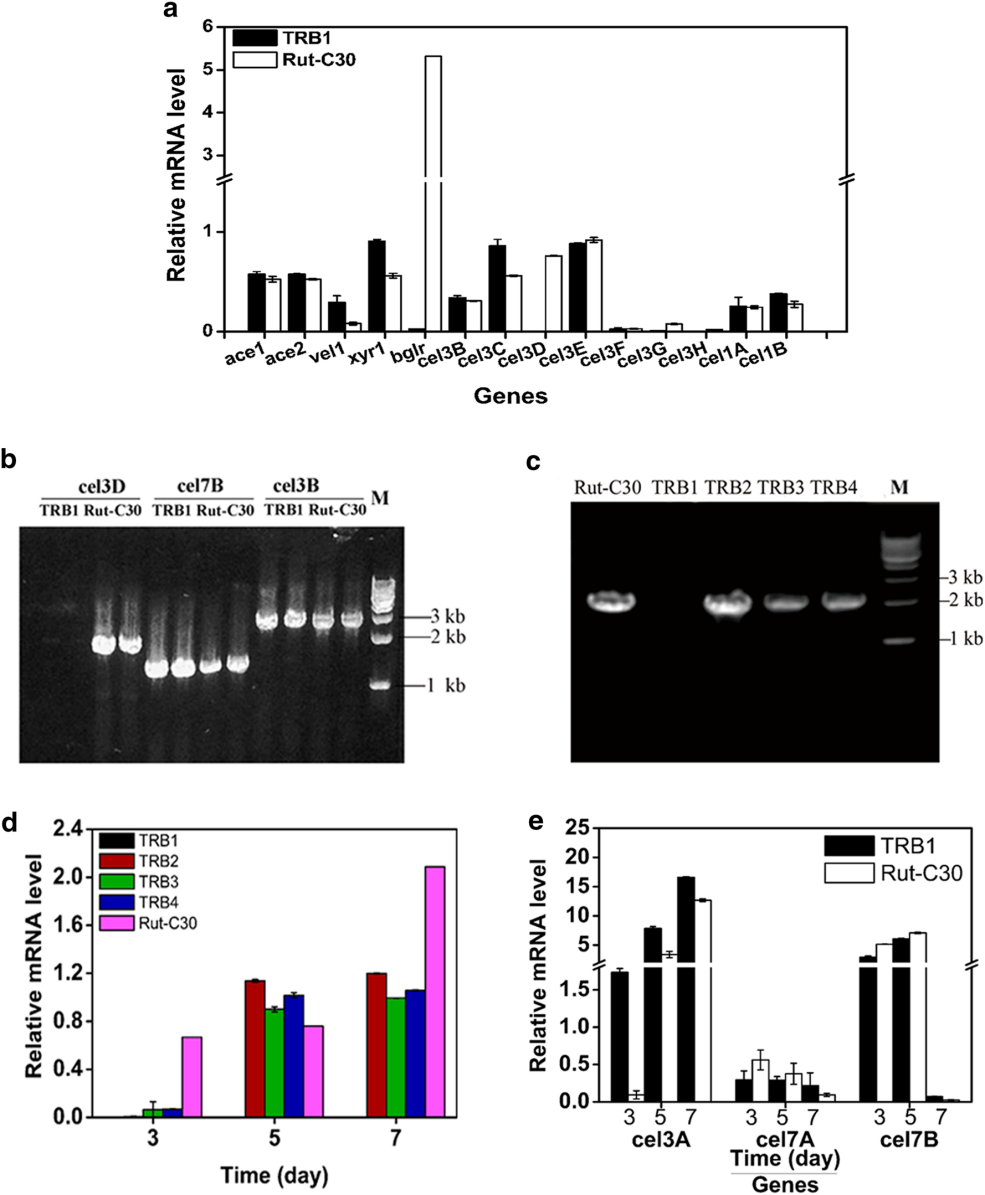
**a** qRT-PCR analysis of the transcript abundance of genes encoding β-glucosidases and transcriptional factors in* T. reesei* TRB1 and RUT-C30 grown on 2 % microcrystalline cellulose for 5 days. **b** Gene clone of cel3D in strain RUT-C30 and the recombinant strains: TRB1, TRB2, TRB3 and TRB4. M: DNA marker; **c** Gene clone of cel3D, cel7B and cel3B in strain Rut-C30 and TRB1; **d** qRT-PCR analysis of the transcript abundance of gene cel3D in strain Rut-C30 and the recombinant strains: TRB1, TRB2, TRB3 and TRB4. **e** qRT-PCR analysis of the transcript abundance of cellulase gene expression in strain RUT-C30 and TRB1. The values show the mean of three replicates, and the *error bar* indicates the standard deviation, though in some cases they are too small to see on the graphical scale being used

In *T. reesei* TRB1, the expression of *T. reesei* bgl1 is probably not the cause of cellulase hyper-production, because other four recombinant *T. reesei* strains with the expression of *T. reesei bgl1* gene did not show cellulase hyper-production (Fig. 1). Hence, we analyzed the transcription of genes related to cellulase production, including cellulase transcription activator ace2 and xyr1, cellulase transcription repressor ace1, the β-glucosidase transcription factor bglR, the global regulator vel1 (essential for cellulase production) [25], and other 9 β-glucosidases (cel1A, cel1B, cel3B, cel3D, cel3C, cel3E, cel3F, cel3G and cel3H) on day 5 by qRT-PCR (Fig. 7a). The transcriptional levels of all the assayed genes in *T. reesei* TRB1 were not affected when compared to *T. reesei* RUT-C30, except vel1, xyr1, bglR, cel3C and cel3D. The transcription abundance of vel1, xyr1 and cel3C in *T. reesei* TRB1 was slightly upregulated, while the mRNA expression of bglR was increased remarkably in TRB1. Interestingly enough, the mRNA expression of cel3D was not detected in *T. reesei* TRB1 during the whole cellulase production process. It seems that the expression of cel3D was completely inhibited in TRB1. We assumed that the open-reading frame (ORF) of cel3D was modified during the random insertion of bgl1 expression cassette mediated by AMT. To prove this, the open-reading frame of cel3D was cloned by PCR using DNA template from both *T. reesei* RUT-C30 and TRB1 (Fig. 7b). There was PCR product of cel3D for RUT-C30, but not for TRB1. As positive controls, the ORFs of both cel3B and cel7B were successfully cloned in both strains. This demonstrated that the open-reading frame (ORF) of cel3D was indeed mutated in TRB1, leading to the undetected mRNA expression of cel3D in TRB1. On the other hand, not only was the ORF of cel3D successfully cloned in other recombinant strains TRB2, TRB3 and TRB4 (Fig. 7c), but also the transcription level of cel3D was detected in these mutant strains during the whole cellulose production (Fig. 7d). These results together suggested that the absence of cel3D in TRB1 is probably involved in its cellulase hyper-production and cel3D may be a repressor of cellulase in RUT-C30.

qRT-PCR analysis of the transcriptional levels of the major extracellular β-glucosidase BGL1 (cel3A), the major cellobiohydrolase CBHI (cel7A), the major endoglucanase CMC (cel7B) in both strain TRB1 and RUT-C30 was performed to determine whether the transcript levels of major cellulolytic enzyme genes were influenced in TRB1 that exhibits cellulase hyper-production and lacks expression of gene cel3D (Fig. 7e). The transcriptional level of cel3A (BGL1) was significantly increased in TRB1, showing greater increase on day 3 (19-fold) and day 5 (2.4-fold) than on day 7 (1.3-fold). As compared with RUT-C30, the bgl1 transcription level in other recombinant strains TRB3 and TRB4 was elevated 1.6- and 2.1-fold respectively (Additional file 1: Figure S1), sharing a similar increase with that in TRB1, but TRB2 and TRB3 did not exhibit β-glucosidase hyper-production. This result demonstrated that a marked expansion of the bgl1 transcription level is not enough to endow the recombinant strains with the phenotype of β-glucosidase hyper-production, supporting that the disruption of cel3D in strain TRB1 might play a part in the excellent performance of strain TRB1 for β-glucosidase production. For reasons unknown, the bgl1 transcription level in strain TRB2 was not affected noticeably (Additional file 1: Figure S1). There is no obvious difference for the transcription level of cel7A (CBHI)and cel7B (CMC) between TRB1 and RUT-C30 during the whole cellulase production process, which is contradictory to the finding that TRB1 showed a moderate increase of both the endoglucanase (EG) activity and the exoglucanase (CBH) activity. These results indicate that the increased cellulase production in strain TRB1 is not mainly resulted from the upregulated transcriptional level of the corresponding genes.

## Discussion

Several advantages were observed in the recombinant strain *T. reesei* TRB1 which we constructed in this study over the parental *T. reesei* RUT-C30, including a significant enhancement of the pNPGase activity, a moderate increase of both the CMCase activity and the pNPCase activity, a minor increment of the FPase activity, and a rapid cellulase induction. These advantages make TRB1 economic feasible and desirable in industrial application, since producing cellulase with all the components in optimal amounts in a shorter period will reduce the cost and improve the efficiency of *T. reesei* cellulase. Although extensive genetic research has been performed to increase the extracellular β-glucosdiase activity in *T. reesei* [8–12], such research only focused on the improvement of β-glucosidase activity [8, 9, 11, 12]. In TRB1, not only the extracellular β-glucosidase activity was increased remarkably, but also the activities of CBH and EG were improved. The β-glucosdiase activity of 18 IU/mL produced in TRB1 was less than the highest β-glucosdiase activity of 30 IU/mL in *T. reesei* reported in the literature [8]. But we’d like to point out that the highest β-glucosdiase activity of 34 IU/mL was obtained using the mixed carbon sources (2 % wheat bran and 3 % microcrystalline cellulose) [8], which are more complex and richer than the single carbon source (2 % microcrystalline cellulose) we utilized here. The mixed carbon sources also endowed the wildtype strain RUT-C30 with 4.4 IU/mL β-glucosdiase activity [8], much higher than 0.7 IU/mL β-glucosdiase activity we got for RUT-C30 under the culture conditions in this study (Fig. 2a) and suggesting that the different carbon sources might give rise to the gap of the beta-glucosidase activity between the literature and this study.

Accordingly, improvement of the saccharification ability of TRB1 cellulase on pretreated corn stover and cellulose was observed (Fig. 5), although the total filter paper activity in TRB1 was only increased slightly (Fig. 2d). Only the three major cellulolytic components: CBH, EG and BGL, are not enough for high efficiency of the cellulose hydrolysis, which was demonstrated by the low cellulose hydrolysis yield from the reconstituted mixture consisting of CBH, EG and BGL [2]. Low abundant essential enzymes in the supernatant of *T. reesei* culture are also important for the high efficiency of *T. reesei* cellulase. These enzymes were possibly reduced in TRB1, considering that the total protein production of TRB1 is not changed, as compared to RUT-C30 (Fig. 2e). This restricts the improvement of total filter paper activity in TRB1. A mixture of TRB1 cellulase and RUT-C30 cellulase might compensate for each other’s enzyme deficiency, resulting higher cellulase efficiency of *T. reesei* cellulase. Such research is undergoing in our lab by testing the saccharification ability of both the cellulase mixture from *T. reesei* RUT-C30 and TRB1, and the cellulase production by co-culturing *T. reesei* RUT-C30 and TRB1.

Deletion of cel1A (BGL2) [13], cel1B [14], and cel3A (BGL1) [24] individually in *T. reesei* has been reported to cause the delay in the induction of cellulase genes by cellulose, while engineered *T. reesei* strains with the overexpression of an thermotolerant exotic β-glucosidase exhibited higher β-glucosidase and total cellulase activities within a shorter incubation time (24 h) compared to the parental strain [12]. It seems that the β-glucosidase activity is related positively to the time of cellulase induction. The higher the β-glucosidase activity is, the faster the cellulase induction is, which is further confirmed by our result that a faster cellulase induction was observed along with the remarkable enhancement of β-glucosidase production in TRB1 (Fig. 2).

The performance of *T. reseei* mutants often relies heavily on what kind of carbon source is available for them and sometimes various results were obtained when different carbon sources were utilized [14, 22]. In this study, the superiority of *T. reesei* TRB1 over RUT-C30 was observed when using cellulose, cellobiose and lactose as the carbon source individually for the cellulase production, indicating that the excellent performance of TRB1 is not dependent on carbon source. Furthermore, *T. reesei* TRB1 showed better resistance to carbon catabolite repression than RUT-C30. TRB1 displays great cellulase production on cellulose even in the presence of 3 % glucose. These merits of TRB1 might benefit the co-culture of *T. reesei* with other microorganisms for valuable biomass-based products where high glucose concentration in the media would increase the substrate flow to these microorganisms and subsequently increase the productivity [25]. Meanwhile, we surprisingly discovered that 1, 2 and 3 % glucose can stimulate slightly the activity of cellulases especially BGL1 in TRB1, which was not observed in RUT-C30. In the previous study, the stimulation of the activity of BGLs of the glycolysis family 1 by glucose has been reported [26], but not BGLs of family 3.

Understanding the molecular mechanism underlying the performance of strain TRB1 could help unravel the regulation mechanism of cellulase production in *T. reesei*. Characterization of secretome of TRB1, and transcription analysis of genes related with cellulase production were performed to identify key factors contributing to the better performance of TRB1 over RUT-C30. According to the SDS-PAGE and MUG-zymogram analysis, the amount of CBH, EG and BGL in the supernatant of *T. reesei* indeed increased along with the enhanced activities of these three enzymes. However, no significant upregulation was observed with the mRNA expression of CBH and EG, with the exception of cel3A. The seemingly conflicting results between the protein expression level and mRNA expression level are normal, since many other factors can influence protein expression such as the mRNA lifetime, the protein degradation rate, etc. The transcription level of the well-known cellulase transcription factors ace2, xyr1, ace1, and vel1 in TRB1 was not affected significantly in comparison with that in RUT-C30. In the same way, the mRNA of all the β-glucosidase genes tested in this study was not affected in TRB1, except for cel3D that was mutated by the random insertion by AMT and was not expressed in TRB1. The lack of cel3D expression in TRB1 might be related to the cellulase hyper-production of TRB1, implying that cel3D might play a part on cellulase production, especially on extracellular β-glucosidase production in *T. reesei*. cel3D, a putative intracellular β-glucosidase, was expressed at a lower level in a *T. reesei* mutant with the deletion of β-glucosidase regulator bglR than the parental strain PC-3-7, when cellobiose was used as the carbon source [20]. The purified cel3D that was heterologously expressed in *E.coli* displayed very low enzyme activity towards cellobiose and other oligosaccharides [15]. Clearly, very few studies have been performed on cel3D and the role of cel3D on the cellulase production remains unexplored. Although studies on other β-glucosidases show that β-glucosidase in *T. reesei* is involved in cellulase induction and production [6, 17, 19, 20], further investigations are required to understand whether and how in TRB1 the lack of cel3D is correlated to the remarkable increase of extracellular β-glucosidase production.

The random insertion of a certain gene into chromosome for overexpression is a common strategy that people harness to study the gene function or improve cellulase production in *T. reesei*. This random insertion is possible to interfere or disrupt other genes’ expression and in turn affect the performance of *T. reseei* in addition to the overexpression of the gene. Hence, different recombinant strains with the overexpression of the same gene might display varied performance, which was observed in our study. However, this effect in most cases has not been taken into account for the performance of the mutant strains in previous studies [8–12]. The effect of the insertion position should definitely be considered when dealing with gene modification by random integration into chromosome in the future.

## Conclusions

The *T. reesei* recombinant strain TRB1 constructed in this study should be more desirable for industrial applications than the parental strain RUT-C30 because it shows extracellular β-glucosidase hyper production, high cellulase production within a shorter time, and a better resistance to carbon catabolite repression. Disruption of β-glucosidase cel3D in TRB1 was identified, which might link to the better performance of TRB1 than RUT-C30 and act in the cellulase production. We suggested that *T. reesei* TRB1 would serve as a great platform to study the role of cel3D on extracellular β-glucosidase production and cellulase production.

## Methods

### Strains, plasmids and culture conditions

Plasmid construction and propagation was performed in *Escherichia coli* DH5α. *Agrobacterium tumefaciens* AGL-1 was used as a T-DNA donor for *T. reesei* transformation by *Agrobacterium tumefaciens*-mediated transformation (AMT) [27]. *T. reesei* RUT-C30 (CICC 13052) was utilized as a parental strain for transformation. *Escherichia coli* and *Agrobacterium tumefaciens* were cultivated in LB medium with 220 rpm at 37 and 28 °C, respectively. *T. reesei* is grown on potato dextrose agar (PDA) plate for conidia culture and in *Trichoderma* minimal media (TMM) [25] with 2 % (w/t) avicel or other carbon sources as indicated in the context for cellulase production at 28 °C with 200 rpm. Plasmid pDHt/sk was provided friendly by Professor Zhihua Zhou from Key Laboratory of Synthetic Biology, Shanghai [21]. Bgl1 gene was amplified from *T. reesei* RUT-C30 cDNA with primers for amplication of bgl1 listed in Additional file 1: Table S1. All chemicals used in this study were purchased from Sigma-Aldrich (St. Louis, MO, USA).

### Construction of recombinant *T. reesei* strains

The total RNA of *T. reesei* RUT-C30 was extracted with the RNA extraction Kit (Omega Bio-Tek, Inc, USA) following the manufacturer’s protocol. The first-strand cDNA was synthesized from RNA using HiScript 1st Strand cDNA Synthesis Kit (Vazyme, Nanjing, China). Bgl1 gene was amplified from *T. reesei* RUT-C30 cDNA with primers for amplication of bgl1 listed in Additional file 1: Table S1, and cloned into the backbone plasmid pDht/sk at XbaI using the ClonExpress One Step Cloning Kit (Vazyme, Nanjing, China), generating plasmid pBGL. Plasmid pBGL was transformed into *T. reesei* RUT-C30 by AMT method [27]. After transferring the putative transformants on PDA medium containing hygromycin B successive for five generations, single spore colonies were isolated and five recombinant *T. reesei* strains were obtained: TRB1, TRB2, TRB3, TRB4, and TRB5, which is confirmed by PCR.

### Shake flask cultivation

To induce cellulase production, the conidial suspension (0.5 mL, 10^7^/mL) was inoculated into a 50 mL Erlenmeyer flask containing 10 mL sabouraud dextrose broth (SDB) and incubated for 48 h with 200 rpm at 28 °C. The culture was then transferred into a 50 mL flask containing 10 mL TMM media (pH 6) [19] with 2 % (w/t) avicel or other carbon sources as indicated in the context for cellulase production at 28 °C with 200 rpm. The TMM media was prepared as follows (all concentrations in g/L unless otherwise noted): urea, 1.00; (NH_4_)_2_SO_4_, 4.00; KH_2_PO_4_, 6.59; K_2_HPO_4_, 1.15; FeSO_4_·7H_2_O, 0.005; MnSO_4_·H_2_O, 0.0016; ZnSO_4_·7H_2_O, 0.0014; CoCl_2_·6H_2_O, 0.002; MgSO_4_, 0.60; CaCl_2_, 0.60; Tween-80, 0.0186 % (v/v); 2.00 % (w/t) avicel or other carbon sources as indicated in the context [25].

### Enzyme assay

Fermentation broth was centrifuged to remove *T. reesei* cells and other solid materials. Culture supernatant was diluted properly for enzyme assays. All enzyme activities were presented as specific activities using international units (IU) per mL supernatant. One IU was defined as the amount of enzyme required to liberate 1 μmol of product per minute under the standard assay conditions. Protein concentration was determined using commercial Bio-Rad protein assay kit which is based on the method of Bradford using bovine serum albumin as a standard. The FPase (FPA) activity and endoglucanase (EG) activity were measured by the DNS method with glucose as a standard, as described in [28, 29]. The β-glucosidase activity was determined using* p*-Nitrophenyl-β-D-glucopyranoside (pNPG) as a substrate based on the reported method by Takashima [30] and Ma [8]. The properly diluted supernatants (20 μL) were incubated with 90 μL of 4 mM pNPG dissolved in 50 mM acetate buffer (pH 5.0) at 50 °C for 10 min. Then, 100 μL of each sample was transferred to 96-well microplate wells, followed by addition of 100 μL 2 % sodium carbonate. The absorbance was measured at 405 nm on a Multiscan FC microplate reader (Thermo Fisher, Shanghai). The exo-1,4-β-glucanase (CBH) activity was measured as reported by Deshpande [31] and Ma [8]. The properly diluted supernatants (20 μL) were mixed with 90 μL 4 mM pNPC dissolved in 50 mM acetate buffer (pH 5.0) containing 1 mg/mL d-glucono-1, 5-σ-lactone, and incubated at 50 °C for 30 min. Then, 100 μL of each sample was transferred to 96-well microplate wells containing 100 μL 2 % sodium carbonate solution. The absorbance was detected at 405 nm on a Multiscan FC microplate reader (Thermo Fisher, Shanghai).

### Preparation of ethylenediamine-pretreated corn stover (PCS) and biomass saccharification

Corn stover was harvested in suburb of Tianjin (China), air-dried, milled and passed through a 2 mm sieve before pretreatment. The moisture of the milled corn stover was 4 %. The milled corn stover was pretreated by ethylenediamine (EDA) in a vacuum drying oven as previous described [32, 33]. EDA pretreatment conditions used for this study include: EDA to biomass loading = 1.0 mL EDA/g dry biomass, temperature 130 °C, residence time 20 min and drying time 60 min. After pretreatment, the pretreated solid was post-washed for three times using total 30 mL water per g solid. The solid was dried at room temperature until the moisture was less than 10 %. The compositions of the pretreated corn stover (PCS) were determined following the Laboratory Analytical Procedure (LAP) of the National Renewable Energy Laboratory (NREL). We found that PCS has 44.8 % glucan, 20.3 % xylan and 4.8 % acid-insoluble lignin in dry matter.

PCS and microcrystalline cellulose were used as the substrates for biomass saccharification according to the reported method by Cheng with slight modifications [34]. Briefly, 10 % (w/v) substrate in 1.5 mL buffer (50 mM sodium citrate buffer at pH 5.0 with 1 mM sodium azide to prevent microbial contamination) and 34 μl crude enzyme were mixed and incubated at 50 °Cwith 400 rpm for 72 h. For a control sample, the crude enzyme was replaced with the buffer. Samples were taken every 24 h and subjected to determination of the reducing sugar level in the supernatant by DNS method.

### SDS-PAGE and MUG-zymogram analysis

SDS-PAGE analysis was carried out on 10 % Tris–HCl polyacrylamide gels using culture supernatants from day 7 with an equal protein concentration of 30 μg for each sample. Proteins were stained by Coomassie blue stain reagent. In-gel β-glucosidase activity was detected by MUG-zymogram analysis. Proteins from the culture supernatants were analyzed by native PAGE using 8 % separation gel and 5 % stacking gels as described by Dashtban [11]. Electrophoresis was run at a constant current of 25 mA at 4 °C for 3 h under non-reducing conditions. After electrophoresis, the gel was washed twice with MiliQ water and incubated with the substrate 4-methylumbelliferyl-β-D-glucopyranoside (MUG, Sigma-Aldrich, USA) in 50 mM sodium citrate buffer (pH 5.0) for 30 min at 50 °C. The fluorescent reaction product was visualized under UV light.

### RNA preparation and real-time quantitative PCR

Fresh mycelia of *T. reesei* TRB1 and *T.reesei* RUT-C30 cultivated under different conditions were prepared and the total RNA was extracted with the RNA extraction Kit (Omega Bio-Tek, Inc, USA) following the manufacturer’s protocol. The first-strand cDNA was synthesized from RNA using HiScript 1st Strand cDNA Synthesis Kit (Vazyme, China). Reverse transcription (RT) was performed using the AceQ qPCR SYBR Green Master Mix (Takara, Dalian, China). The reversed RNA concentration was determined at 260 nm using a NanoDrop ND-2000 (Thermo Fisher Scientific, Wilmington, DE). qRT-PCR was performed on the ABI StepOne instrument Plus (ABI, Germany) with software Version 2.3 (ABI, Germany). The primers for qRT-PCR are shown in Additional file 1: Table S1. At least three biological triplicates were performed, and qRT-PCR of each gene was performed in three triplicates. The expression of pgk1 was chosen as the reference gene for data normalization.

## Additional file

10.1186/s12934-016-0550-3 qRT-PCR analysis of the transcript abundance of gene bgl1 in the recombinant *T. reesei* strains (TRB1-4) and RUT-C30 grown on 2 % microcrystalline cellulose for 5 days. **Table S1.** PCR primers used for plasmid construction and real-time quantitative PCR.

## Abbreviations

pNPGase: the β-glucosidase activity
pNPCase: the CBH activity
CMCase: the CMC activity
FPase: the filter paper activity
AMT: agrobacterium tumefaciens-mediated transformation
PDA: potato dextrose agar
SDB: sabouraud dextrose broth
TMM: *Trichoderma* minimal medium
IU: international units
pNPG: *p*-nitrophenyl-β-d-glucopyranoside
EDA: ethylenediamine
PCS: the pretreated corn stover
MUG: 4-methylumbelliferyl-β-d-glucopyranoside
CCR: carbon catabolite repression
ORF: open-reading frame

## References

[1] Kubicek CP, Mikus M, Schuster A, Schmoll M, Seiboth B (2009). Metabolic engineering strategies for the improvement of cellulase production by *Hypocrea jecorina*. Biotechnol Biofuels.

[2] Adav SS, Chao LT, Sze SK (2012). Quantitative secretomic analysis of *Trichoderma reesei* strains reveals enzymatic composition for lignocellulosic biomass degradation. Mol Cell Proteomics.

[3] Cherry JR, Fidantsef AL (2003). Directed evolution of industrial enzymes: an update. Curr Opin Biotechnol.

[4] Saloheimo M, Kuja-Panula J, Ylösmäki E, Ward M, Penttilä M (2002). Enzymatic properties and intracellular localization of the novel *Trichoderma reesei* β-glucosidase BGLII (Cel1A). Appl Environ Microbiol.

[5] Karkehabadi S, Helmich KE, Kaper T, Hansson H, Mikkelsen NE, Gudmundsson M, Piens K, Fujdala M, Banerjee G, Scott-Craig JS (2014). Biochemical characterization and crystal structures of a fungal family 3 beta-glucosidase, Cel3A from *Hypocrea jecorina*. J Biol Chem.

[6] Berlin A, Maximenko V, Gilkes N, Saddler J (2007). Optimization of enzyme complexes for lignocellulose hydrolysis. Biotechnol Bioeng.

[7] Treebupachatsakul T, Nakazawa H, Shinbo H, Fujikawa H, Nagaiwa A, Ochiai N, Kawaguchi T, Nikaido M, Totani K, Shioya K (2016). Heterologously expressed *Aspergillus aculeatus* β-glucosidase in *Saccharomyces cerevisiae* is a cost-effective alternative to commercial supplementation of β-glucosidase in industrial ethanol production using *Trichoderma reesei* cellulases. J Biosci Bioeng.

[8] Ma L, Zhang J, Zou G, Wang C, Zhou Z (2011). Improvement of cellulase activity in *Trichoderma reesei* by heterologous expression of a beta-glucosidase gene from *Penicillium decumbens*. Enzyme Microbial Technol.

[9] Nakazawa H, Kawai T, Ida N, Shida Y, Kobayashi Y, Okada H, Tani S, Sumitani J, Kawaguchi T, Morikawa Y (2012). Construction of a recombinant *Trichoderma reesei* strain expressing* Aspergillus aculeatus *beta-glucosidase 1 for efficient biomass conversion. Biotechnol Bioeng.

[10] Treebupachatsakul T, Shioya K, Nakazawa H, Kawaguchi T, Morikawa Y, Shida Y, Ogasawara W, Okada H (2015). Utilization of recombinant *Trichoderma reesei* expressing* Aspergillus aculeatus* beta-glucosidase I (JN11) for a more economical production of ethanol from lignocellulosic biomass. J Biosci Bioeng.

[11] Dashtban M, Qin W (2012). Overexpression of an exotic thermotolerant beta-glucosidase in *Trichoderma reesei* and its significant increase in cellulolytic activity and saccharification of barley straw. Microb Cell Fact.

[12] Zhang J, Zhong Y, Zhao X, Wang T (2010). Development of the cellulolytic fungus *Trichoderma reesei* strain with enhanced beta-glucosidase and filter paper activity using strong artificial cellobiohydrolase 1 promoter. Bioresour Technol.

[13] Saloheimo M, Kuja-Panula J, Ylosmaki E, Ward M, Penttila M (2002). Enzymatic properties and intracellular localization of the novel *Trichoderma reesei* beta-glucosidase BGLII (cel1A). Appl Microbiol Biotechnol.

[14] Zhou Q, Xu J, Kou Y, Lv X, Zhang X, Zhao G, Zhang W, Chen G, Liu W (2012). Differential involvement of beta-glucosidases from *Hypocrea jecorina* in rapid induction of cellulase genes by cellulose and cellobiose. Eukaryot Cell.

[15] Guo B, Sato N, Biely P, Amano Y, Nozaki K (2016). Comparison of catalytic properties of multiple β-glucosidases of *Trichoderma reesei*. Appl Microbiol Biotechnol.

[16] Fowler T, Brown RD (1992). The bgI1 gene encoding extracellular β-glucosidase from *Trichoderma reesei* is required for rapid induction of the cellulase complex. Mol Microbiol.

[17] Xu J, Zhao G, Kou Y, Zhang W, Zhou Q, Chen G, Liu W (2014). Intracellular beta-glucosidases CEL1a and CEL1b are essential for cellulase induction on lactose in *Trichoderma reesei*. Eukaryot Cell.

[18] Porciuncula Jde O, Furukawa T, Shida Y, Mori K, Kuhara S, Morikawa Y, Ogasawara W (2013). Identification of major facilitator transporters involved in cellulase production during lactose culture of *Trichoderma reesei* PC-3-7. Biosci Biotechnol Biochem.

[19] Shida Y, Yamaguchi K, Nitta M, Nakamura A, Takahashi M, Kidokoro S, Mori K, Tashiro K, Kuhara S, Matsuzawa T (2015). The impact of a single-nucleotide mutation of bgl2 on cellulase induction in a *Trichoderma reesei* mutant. Biotechnol Biofuels.

[20] Nitta M, Furukawa T, Shida Y, Mori K, Kuhara S, Morikawa Y, Ogasawara W (2012). A new Zn(II)(2)Cys(6)-type transcription factor BglR regulates beta-glucosidase expression in *Trichoderma reesei*. Fungal Genet Biol.

[21] Zou G, Shi S, Jiang Y, van den Brink J, de Vries RP, Chen L, Zhang J, Ma L, Wang C, Zhou Z (2012). Construction of a cellulase hyper-expression system in *Trichoderma reesei* by promoter and enzyme engineering. Microb Cell Fact.

[22] Shida Y, Yamaguchi K, Nitta M, Nakamura A, Takahashi M, Kidokoro SI, Mori K, Tashiro K, Kuhara S, Matsuzawa T (2015). The impact of a single-nucleotide mutation of bgl2 on cellulase induction in a *Trichoderma reesei* mutant. Biotechnol Biofuels.

[23] Le Crom S, Schackwitz W, Pennacchio L, Magnuson JK, Culley DE, Collett JR, Martin J, Druzhinina IS, Mathis H, Monot F (2009). Tracking the roots of cellulase hyperproduction by the fungus *Trichoderma reesei* using massively parallel DNA sequencing. Proc Natl Acad Sci.

[24] Fowler T, Brown RD (1992). The bgI1 gene encoding extracellular β-glucosidase from Trichoderma reesei is required for rapid induction of the cellulase complex. Mol Microbiol.

[25] Minty JJ, Singer ME, Scholz SA, Bae C-H, Ahn J-H, Foster CE, Liao JC, Lin XN (2013). Design and characterization of synthetic fungal-bacterial consortia for direct production of isobutanol from cellulosic biomass. Proc Natl Acad Sci USA.

[26] Antonieto ACC, Castro LD, Silva-Rocha R, Persinoti GF, Silva RN (2014). Defining the genome-wide role of CRE1 during carbon catabolite repression in *Trichoderma reesei* using RNA-Seq analysis. Fungal Genet Biol.

[27] Zhong YH, Wang XL, Wang TH, Jiang Q (2007). Agrobacterium-mediated transformation (AMT) of *Trichoderma reesei* as an efficient tool for random insertional mutagenesis. Appl Microbiol Biotechnol.

[28] Xiao Z, Storms R, Tsang A (2004). Microplate-based filter paper assay to measure total cellulase activity. Biotechnol Bioeng.

[29] Xiao Z, Storms R, Tsang A (2005). Microplate-based carboxymethylcellulose assay for endoglucanase activity. Anal Biochem.

[30] Takashima S, Nakamura A, Hidaka M, Masaki H, Uozumi T (1999). Molecular cloning and expression of the novel fungal beta-glucosidase genes from Humicola grisea and *Trichoderma reesei*. J Biochem.

[31] Deshpande MV, Eriksson KE, Göran Pettersson L (1984). An assay for selective determination of exo-1,4,-β-glucanases in a mixture of cellulolytic enzymes. Anal Biochem.

[32] Qin L, Li WC, Zhu JQ, Liang JN, Li BZ, Yuan YJ (2015). Ethylenediamine pretreatment changes cellulose allomorph and lignin structure of lignocellulose at ambient pressure. Biotechnol Biofuels.

[33] Qin L, Liu L, Li WC, Zhu JQ, Li BZ, Yuan YJ (2016). Evaluation of soluble fraction and enzymatic residual fraction of dilute dry acid, ethylenediamine, and steam explosion pretreated corn stover on the enzymatic hydrolysis of cellulose. Bioresour Technol.

[34] Cheng Y, Song X, Qin Y, Qu Y (2009). Genome shuffling improves production of cellulase by Penicillium decumbens JU-A10. J Appl Microbiol.

