# Supplementary material for: A β-glucosidase hyper-production Trichoderma reesei mutant reveals a potential role of cel3D in cellulase production
Source: Microb Cell Fact. 2016 Sep 1;15(1):151. doi: 10.1186/s12934-016-0550-3 (PMC5009570; doi:10.1186/s12934-016-0550-3)
Supplement: Supplementary file 1 — 10.1186/s12934-016-0550-3 qRT-PCR analysis of the transcript abundance of gene bgl1 in the recombinant T. reesei strains (TRB1-4) and RUT-C30 grown on 2 % microcrystalline cellulose for 5 days. Table S1. PCR primers used for plasmid construction and real-time quantitative PCR. [file 12934_2016_550_MOESM1_ESM.docx]

Additional file:

| **Target gene** | **Forward primer(5’to 3’)** | **Reverse primer (5’to 3’)** |
| --- | --- | --- |
| **For amplification of bgl1** | | |
| BGL his | ACCCAATAGTCAATCTAGAATGCGTTACCGAACAGCAGC | TCGGCATCTACTTCTAGATTAATGATGATGATGATGATGCGCTACCGACAGAGTGCTCG |
| **For qRT-PCR** | | |
| cel7a | GCGGATCCTCTTTCTCAGAC | TTGGCGTAGTAATCATCCCA |
| cel7b | ACTACACGGAGGAGCTCGACGACTT | AAGGCATTGCGAGTAGTAGTCGTTG |
| cel3a | ATGCGTTACCGAACAGCAGCTGC | TGCGGCCTTCGCCTTGTCGTAC |
| cel3b | CAACCTCACCACCGGTGTTG | AAGCCAACGCTGAAAGCGC |
| cel3c | CCCTGGCCGAGAAGGTCGA | AGCAGGCCGCAGGGACGC |
| cel3d | GCGAGGATGTTTACGTTGGCTAC | TGGGCTGGTCCGGGTGGA |
| cel3e | TCTTGGGGCAAGAATCTGACAG | CTGTTTCAAGCCGATGCCC |
| ace1 | AAGACCCTGATCTTCATGGC | ATTCGACTGTCGCTTGAATG |
| ace2 | GCAGCAGCTGAGAGAGTACG | ATAGAGGGAGGCGAGATCCT |
| cel1a | CCTACCAGATCGAGGGCGC | GCAGCGCAATGTCCTCGG |
| cel1b | TCGCACTTGGACTCGATTTCC | CTTGAGGGTGGTGTAGTCTGTGAAC |
| vel1 | ATCGCGTGACGAGAGAGAAC | ATGCAGGAACACCAGTCAGG |
| xyr1 | TACCAAGTGCGATGGCTTAC | CTCTCTCGGACATATTCGCA |

**Table S1** PCR primers used for plasmid construction and real-time quantitative PCR





Figure S1 qRT-PCR analysis of the transcript abundance of gene bgl1 in the recombinant *T. reesei* strains (TRB1-4) and RUT-C30 grown on 2% microcrystalline cellulose for 5 days.
